# Multi-task learning to leverage partially annotated data for PPI interface prediction

**DOI:** 10.1038/s41598-022-13951-2

**Published:** 2022-06-21

**Authors:** Henriette Capel, K. Anton Feenstra, Sanne Abeln

**Affiliations:** 1 Bioinformatics Section VU, Vrije Universiteit Amsterdam, 1081HV Amsterdam, The Netherlands; 2 Life Science and Health, CWI, Amsterdam, The Netherlands

**Keywords:** Machine learning, Protein function predictions

## Abstract

Protein protein interactions (PPI) are crucial for protein functioning, nevertheless predicting residues in PPI interfaces from the protein sequence remains a challenging problem. In addition, structure-based functional annotations, such as the PPI interface annotations, are scarce: only for about one-third of all protein structures residue-based PPI interface annotations are available. If we want to use a deep learning strategy, we have to overcome the problem of limited data availability. Here we use a multi-task learning strategy that can handle missing data. We start with the multi-task model architecture, and adapted it to carefully handle missing data in the cost function. As related learning tasks we include prediction of secondary structure, solvent accessibility, and buried residue. Our results show that the multi-task learning strategy significantly outperforms single task approaches. Moreover, only the multi-task strategy is able to effectively learn over a dataset extended with structural feature data, without additional PPI annotations. The multi-task setup becomes even more important, if the fraction of PPI annotations becomes very small: the multi-task learner trained on only one-eighth of the PPI annotations—with data extension—reaches the same performances as the single-task learner on all PPI annotations. Thus, we show that the multi-task learning strategy can be beneficial for a small training dataset where the protein’s functional properties of interest are only partially annotated.

## Introduction

**Figure 1:**
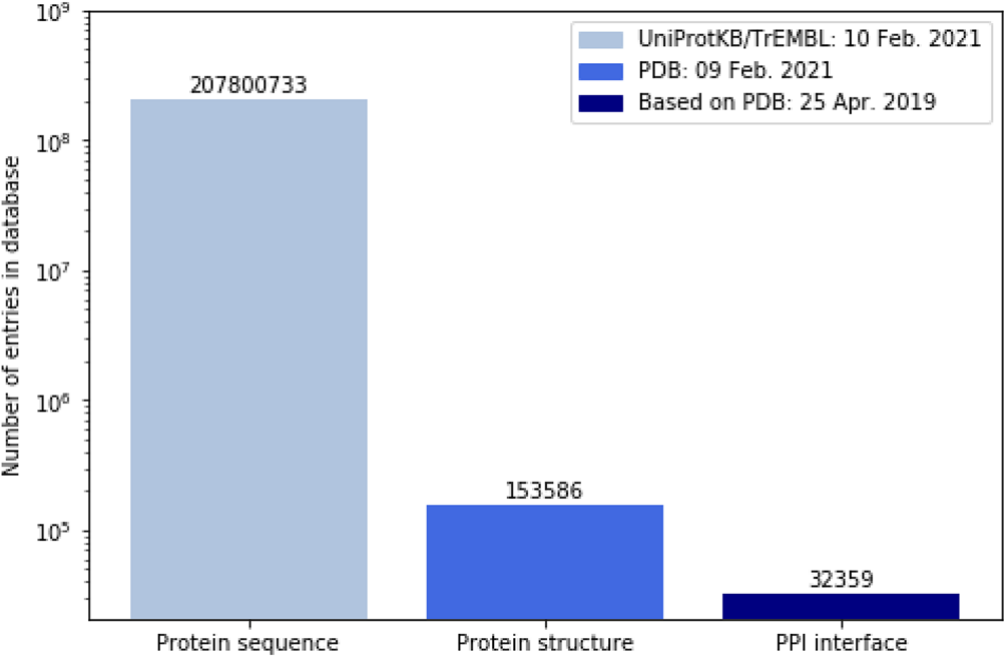
Comparison of the number of entries available in databases on protein sequence, protein structure and specific structure-based functional annotations: protein–protein interaction (PPI) interface. These results are respectively based on the protein entries available in the UniProtKB/TrEMBL database, protein entries available in the Protein Data Bank (PDB), and the protein entries with PPI interface annotations. Note that the y-axis is logarithmic.

Protein sequence databases^1^ continue to grow rapidly and structural information is becoming more readily available^2^. Nevertheless, precise functional annotation based on the protein structure, such as protein binding sites^3^, are still scarce, and difficult to predict. Therefore, computational techniques are used to predict several functional structural properties of proteins based on the protein sequence. One of these properties is the physical interaction interface between proteins which are crucial for the functioning of a protein^4^. Interaction between proteins is required in many biological processes, such as DNA replication, RNA transcription, signal transduction, control of cellular processes, protein transport, and metabolism^5–9^. Furthermore, many diseases can be related to the deformation of a protein’s interface^10,11^. Predicting the set of residues in a protein that interact with other proteins is an important, but still challenging task^12^. Moreover, structural information on residues that make up the interface is scarce. The size of the PPI annotated database is only a small fraction of the size of structural annotated database. The size of the structural annotated database, in turn, is a small fraction of the size of the protein sequence database (see Fig. 1). In addition, there are problems such as the prediction of epitopes (antibody-binding) interfaces, for which even less labelled data is available^13^. To efficiently train deep neural networks for the PPI interface prediction, and other tasks little annotation availability, we have to overcome the problem of the limited size of the training dataset.

Due to the successes of deep learning in fields such as natural language processing, deep learning approaches are increasingly used and have shown great successes for protein structural feature prediction^14–17^. In deep learning, multiple connected layers, along with their parameters, predict the output of the corresponding input features^18^. Approaches and models such as convolutional neural networks (CNN), residual neural networks (ResNet), recurrent neural networks (RNN), long short term memory networks (LSTM), transformers, and multi-task learners appear in recent structure prediction methods^15,16,19–22^. Hanson et al.^16^ used, among others, ultra-deep ResNets in the SPOT-1D model which were able to capture non-local interactions between residues that are only close in the protein structure and not in the protein sequence^16^. Heffernan et al.^21^ used LSTM bidirectional RNNs and showed that this method is useful to capture long range interactions, especially for residues with large numbers of long-range contacts. We recently compared the usage of different neural network architectures for the prediction of protein interfaces^23^. Furthermore, transformers have been successfully used in the language of proteins^24,25^. In transformers, information learned from general domain data, like protein sequences, is transferred to domain specific data, such as secondary structure prediction. Another strategy in which information is transferred is multi-task learning.

In contrast to single-task learning, in which the aim is to improve the performance of one specific prediction task, for multi-task learning^26^ the aim is to improve the performance of multiple learning tasks simultaneously. Training the multi-task model on different tasks at the same time allows the model to learn a shared representation, providing a way to transfer information learned between specific tasks^14,26^. Multi-task learning is related to inductive transfer learning^27^. The main difference is that the aim of inductive transfer learning is to only achieve high performances for the main task, whereas the aim of multi-task learning is to learn both the main and related tasks^27^. The multi-task learning strategy can be implemented in the end-to-end learning architecture of deep learning models. In order to learn the model to make accurate predictions for all tasks, the loss of the various tasks should be represented in the loss function used during training the multi-task model^14^. This strategy has previously been applied to the domain of proteins by, for example, concurrently predicting multiple protein structural properties^15^. The benefit of using information on structural annotations, as input features using pretrained prediction models, has already been shown for several single-task learners^7,28,29^. One advantage of the multi-task strategy is not having to generate input features a priori when applying the model to a new input. Here we are interested both in the inductive transfer’s learning ability of the multi-task setup to improve the performance of PPI interface prediction, as well as the actual predictions of the related tasks, such as surface accessibility, as these may provide, for example, insight in the nature of the binding site. Here we investigate if a multi-task learning strategy may be suitable to train PPI interface prediction models.

To consider which *related work* has been performed, we should cover two types of problems: (1) protein structural property prediction in a multi-task setting and (2) protein interface prediction. Protein structural property prediction methods commonly use a multi-task learning strategy. Note that the labels for many structural properties, such as secondary structure and solvent accessibility, can be generated only if the three-dimensional structure of a protein is available. Klausen et al.^15^ built the sequence-based method NetSurfP-2.0 to predict solvent accessibility, secondary structure, structural disorder and backbone dihedral angles, using a combination of CNNs and LSTMs. The deep learning model SPOT-1D is based on an ensemble of ResNets and CNNs to predict secondary structure, backbone angles, solvent accessibility and contact number^16^. Secondary structure prediction by SPOT-1D resulted in higher performances than reached by NetSurfP-2.0^16^. In 2020, Xu et al.^17^ published their method OPUS-TASS. This multi-task learner, based on a combination of CNNs, transformers and LSTMs, was able to improve the predictions of secondary structure and backbone angles even further^17^. Xu et al.^17^ trained multiple models, including a different set of the learning tasks: secondary structure in three and eight classes, backbone torsion angles, absolute solvent accessibility, side-chain dihedral angles, and the local backbone structure descriptor CSF3^17^. The multi-task learning strategy has, to the best of our knowledge, not been used for PPI interface prediction.

PPI prediction models were recently described in the review paper by Savojardo et al.^12^. In this paper the different models were distinguished into methods using the primary protein sequence as input, and methods using the three-dimensional protein structure as input for the prediction model. Moreover, methods can be partner independent or partner dependent^12^. In this study, we predict PPI interface residues based on the primary sequence in a partner-unspecific way. The most recent other sequence-based partner-unspecific models are SSWRF^30^, SeRenDIP^7,31^, SCRIBER^9^, and PIPENN^23^. The SSWRF method uses an ensemble support vector machine and a sample-weighed random forest to predict the PPI interface^30^. SeRenDIP is a random forest model trained on datasets containing either only homomeric interactions, only heteromeric, or containing both types of interactions^7^. SCRIBER is a model based on multilevel logistic regression and trained on a dataset containing multiple types of protein interactions.

Most interface prediction methods use the following features as input: sequence conservation (see below)^8,9,28,29^, surface accessibility^8,9,30,32–34^, backbone flexibility^35,36^ or a combination of these^7,31^ as input features. Previous studies showed that high solvent accessible residues are more likely to be interface residues^32–34^. The train and testing data, to annotate proteins with true binding interfaces can be retrieved from the PDB. However, this is not entirely trivial as one needs to define the interface of the of the binding molecules. Typically, some threshold is used to select amino acids in close proximity^9,23^. Some larger published datasets are available, notably ‘ZK448’, a 448 protein test-set by Zhang and Kurgan^9^, ‘BioDL’ containing in total 4620 proteins with PPI annotations by Stringer et al.^23^, and ‘Homomeric & Heteromeric’ with 546 proteins by Hou et al.^7,31^, which each come with their sets of precalculated features. However, not all datasets include all features, and generation of missing features may be a time-consuming task. State of the art performances for partner-unspecific PPI interface prediction range between 0.68 and 0.78 AUC ROC, depending on the exact dataset and model used^23^. Some types of PPIs are more difficult to predict than others: heteromeric interfaces tend to be more difficult than homomeric interfaces^7^. Performances also typically differ between datasets, and of those mentioned above ZK448 shows lower performance metrics overall^23^. We refer to Stringer et al.^23^, Zhang and Kurgan^9^ and Hou et al.^31^ for recent reviews and benchmarks of these methods, and we will return to benchmark performances in “Discussion”.

Protein sequence conservation profiles provide a very strongly signal for many functional and structural prediction tasks, as they encode which residues were constrained during evolution and are therefore likely to have an important functional or structural role. Note that the highest accuracy for protein structural prediction tasks can only be obtained if conservation is used as an input feature^37^. In fact, even of the state-of-the-art structure prediction models have to use such profiles as input features^2^, and can not fully define an end-to-end problem description from sequence to structure without explicitly calculating conservation profiles. These conservation patterns are typically encoded as Position Specific Scoring Matrix (PSSM) or Hidden Markov Model (HMM) profiles, which provide additional features for each residue. Existing PPI interface prediction methods also use conservation as input^7–9,30,38–40^.

**Figure 2:**
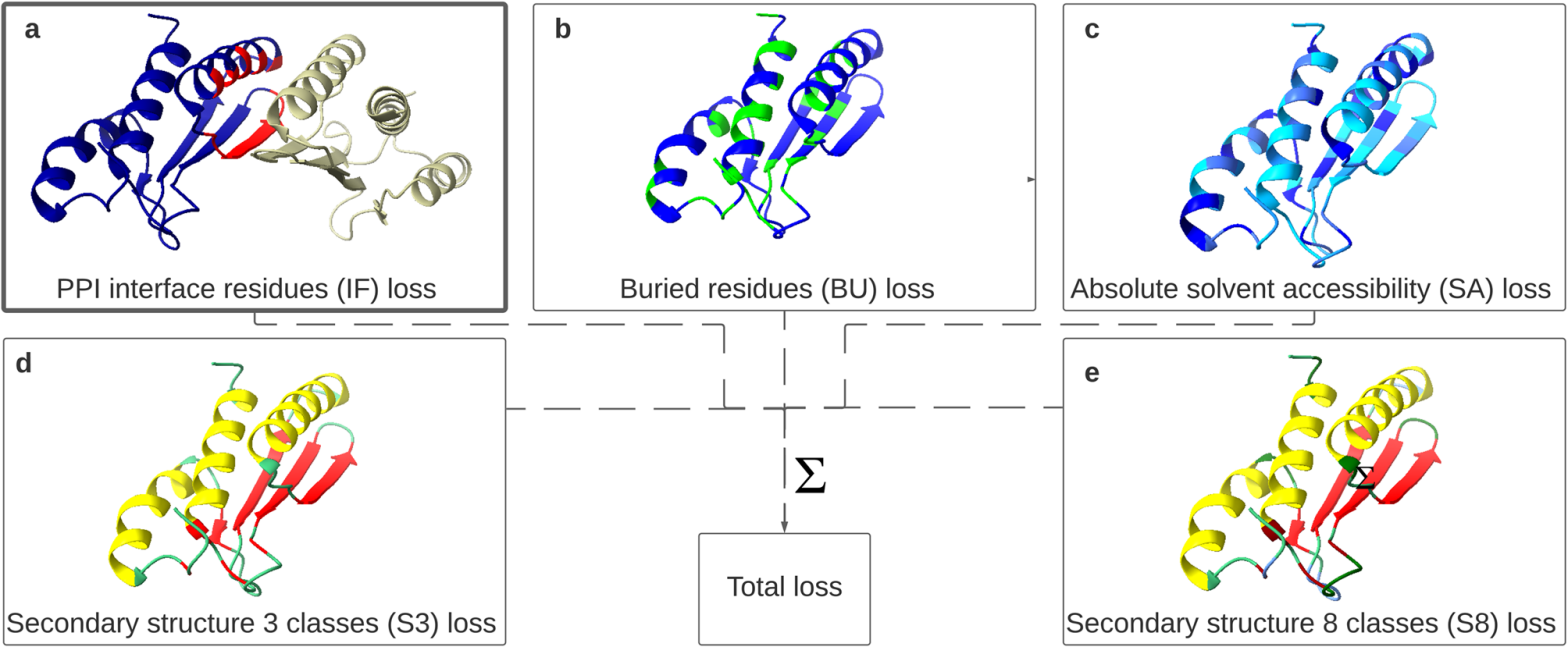
Visualisation of the possible protein structural prediction tasks, and the implementation of the multi-task setup in the cost function of the model. The protein in the example is Pterin-4-alpha-carbinolamine dehydratase 2, based on the PDB structure 4wil chain A. (**a**) The protein–protein interaction interface residues are colored red for the protein chain A, the other residues of chain A are depicted in blue. These residues interact with the olive coloured protein chain. (**b**) Buried residues are indicated in green. (**c**) The absolute solvent accessibility is shown in blue colors. The darker the color of the residues the higher the solvent accessibility. (**d**) Classification of the secondary structure components in three classes. (**e**) Coil is colored green, $\alpha$-helix yellow and $\beta$-strand red. Classification of the secondary structure components in eight classes. Coil is colored green, high-curvature blue, $\beta$-turn dark green, $\alpha$-helix yellow, $\beta$-strand red and $\beta$-bridge dark red. Note that this protein does not have a 3$_{10}$-helix or a $\pi$-helix. The loss of the individual (possible) prediction tasks are summed in the cost function which is used during training of the multi-task model.

In this work, we take sequence-derived properties and sequence-conservation as input features, similar to OPUS-TASS^17^, but do not use predicted structural properties such as secondary structure or surface accessibility as input features—as is common in many PPI interface prediction methods. Instead, these structural properties are used as related learning tasks in our multi-task setup, as shown schematically in Fig. 2.

Here, we investigate if PPI interface prediction—when the size of the training dataset is the limiting factor for performance—can be improved by defining the task as a multi-task learning problem. We show model performances for different combinations of the related learning tasks: secondary structure in three and eight classes, absolute solvent accessibility, and buried residues. Using these tasks, higher prediction performances are reached compared to the single-task interface prediction model. In addition, the multi-task setup offers the possibility of training on a partially annotated dataset by continuing learning on exclusively the related tasks. The dataset used in this study is only partially annotated with PPI interface labels. The benefit of the multi-task set up, as a solution to missing data, is studied here in more depth by masking PPI interface labels for a part of the proteins in our dataset. We show that formulating a predication task as a multi-task learning problem can be beneficial for protein structural prediction tasks for which only a small set of annotated training data is available.

## Methods

The OPUS-TASS model described by Xu et al.^17^ is used as the basis for the prediction models used in this study. We also used their published annotated dataset, and their training and validation procedures.

### Datasets

The combined OPUS-TASS training and validation set consists of 11,007 proteins and includes the following generated input features: HMM profiles, PSSM profiles, physicochemical features, and the PSP19 feature. Note that HMM and PSSM profiles are computationally expensive to generate. The proteins in this dataset were selected by Hanson et al.^41^, and were also used for training and validating SPOT-1D^16^. The proteins are culled from the PISCES^42^ server in February 2017. Only structures that are obtained by X-ray crystallography at a resolution better than 2.5 Å were selected. Sequences exceeding a sequence length of 700 residues were removed and the dataset was filtered by sequence identity, applying a cut-off of 25%. Residues based annotations for PPI interfaces are available for one third of this dataset (3551 proteins). This PPI interface annotated data is a selection of PDB^43^ structures as described by Stringer et al.^23^. In short, the procedure was as follows. Proteins consisting of 2–200 chains were selected. For one structural complex the inter-atomic distance between all amino acids in separate chains were determined. The amino acids were defined as binding residues when the inter-atomic distance falls below a certain threshold. This threshold was set to 0.5 Å plus the van der Waals radii of the two atoms.

Two datasets were constructed to see if we could train the multi-task model effectively using limited data: (1) the ‘PPI dataset’ contains all proteins in the OPUS-TASS dataset for which PPI annotations were available; (2) the extended ‘PPI_extendedSFD dataset’ contains the PPI dataset extended with structural feature data stored in all other proteins of the OPUS-TASS dataset (see Supplementary Fig. 1). The PPI_extendedSFD dataset is therefore larger, but only partially annotated with PPI interface information. Both datasets were split into a training (80%), validation (10%) and test (10%) set. For all these sets the PPI dataset is a strict subset of the PPI_extendedSFD dataset, and thus contains the same PPI interface information. Splitting the data into training, validation, and test sets was performed *after* matching the proteins of OPUS-TASS dataset with PPI annotations based on the PDB ID and protein chain. The PPI annotations for 64 proteins had to be removed because the protein sequences between the two database did not correspond.

### Input features

The sequence-based input features consist of 20 features obtained from the Position Specific Scoring Matrix (PSSM), 30 features obtained from Hidden Markov Model (HMM) profiles, seven features obtained from the physicochemical properties and 19 features obtained from the PSP19 classification. Hence, every protein is represented by a matrix with the following dimensions: the number of protein residues times the 76 input features ($20+30+7+19$). The PSSM profiles, constructed by Xu et al.^17^, are based on three iterations of PSI-BLAST (v2.10.0+)^44^ using the UniRef90 database^45^. The HMM profiles are constructed using HHBlits (v3.1.0)^46^ and the Uniclust30 database^47^. HMMs capture position-specific information about insertions and deletions, additional to conservation, for each amino acid. Only the conservation per amino acid is captured in the PSSM^37^. HHBlits is a fast sequence search algorithm using HMM-HMM alignment after applying a profile-profile alignment as pre-filter^48^. The seven physicochemical properties are the amino acid properties as described by Meiler et al.^49^. The PSP19 feature captures side-chain flexibility and packing orientation^50^. For this feature 19 rigid-body blocks were constructed by Lu et al.^50^ in a one-hot encoded sequence indicating the existence of a block in a protein residue. Both the physicochemical properties and the PSP19 feature are protein independent and amino acid specific.

### Prediction tasks

During pre-processing, the prediction task labels were generated. For our models, we considered three output labels generated by Xu et al.^17^: secondary structure in three and eight classes (S3 and S8), and the solvent accessibility of residues (SA). The three class secondary structure components are coil, $\alpha$-helix and $\beta$-strand. These three components can be further distinguished into eight classes: coil into coil, high-curvature, and $\beta$-turn; $\alpha$-helix into $\alpha$-helix, 310-helix, and $\pi$-helix; and $\beta$-strand into $\beta$-strand and $\beta$-bridge^51^. The S3, S8, and SA labels are derived from DSSP^51^. In addition, we added two classification tasks: identification of buried (BU) and PPI interface (IF) residues. Residues were labelled as buried if the fraction of absolute solvent accessibility over its maximum solvent accessibility is less than 7% (Supplementary Algorithm 1)^52^. Residues with incomplete side chains were masked for the SA prediction. For the PPI labels all residues of a protein were masked when no PPI interface annotation was available for the entire protein. Masked residues were not taken into account in the loss calculation and performance measures. The prediction tasks S3, S8, SA, and BU are related to the PPI interface and therefore used in this study as the possible additional learning tasks for the multi-task IF predictor.

### Model architecture

The details of the deep learning model architecture are described by Xu et al.^17^ in their “Methods” and Fig. 1. The model is implemented in python using the keras library of tensorflow^53^. Similar to their model we performed data enhancement and used the architecture of 2 transformer layers, 5 CNN layers and 4 bidirectional LSTM layers (see Supplementary Fig. 2). We used their dropout of 0.25 and the rectified linear unit (ReLU) activation function. In contrast to the OPUS-TASS model the possible output labels in our models are: interface residues (IF), secondary structure based on 3 classes (S3) and on 8 classes (S8), absolute solvent accessibility (SA), and buried residues (BU). The models studied are named based on the abbreviations of the prediction tasks that are considered in the model. We do not create an ensemble of these different models. Furthermore, we added the possibility to mask part of the PPI interface data (see Supplementary Algorithm 2), to study the effect of partially annotated data.

### Multi-task learning process

The multi-task learning setup is implemented in the cost function of the model. For each prediction task the individual loss is determined by the cross-entropy for classification tasks (S3, S8, BU, IF) and mean squared error for the regression task (SA). The individual losses are summed and form the total cost function, as shown in Fig. 2, and as defined as:

1 $$\begin{aligned} L= \alpha L_{IF} + \beta L_{BU} + \gamma L_{S3} + \delta L_{S8} + \varepsilon L_{SA} \end{aligned}$$

where $L_{IF}$ is the cross-entropy loss for the interface predictions, $L_{S3}$ is the cross entropy loss for the S3 secondary structure predictions, $L_{S8}$ is the cross entropy loss for the S8 secondary structure predictions, $L_{SA}$ is the mean squared error loss for the surface accessibility predictions.

In this way the model is able to learn a shared representation for all the prediction tasks. For the majority of the proteins in the PPI_extendedSFD dataset no PPI interface information is available. For these proteins the loss is constructed solely by the individual losses of the related tasks. We investigated the effect of the relative weights for each task in the cost function, using three approaches. Method A: the weights of the individual learning tasks losses in the total cost function were set equal, i.e. $\alpha = \beta = \gamma = \delta = \varepsilon$. Method B: similar prediction tasks were grouped together (S3, S8 and BU, SA). The weights of pairs of similar prediction tasks were halved, i.e. $\alpha = (\beta + \gamma ) = (\delta + \varepsilon$) and $\beta = \gamma$, $\delta = \varepsilon$ if both prediction tasks associated with the summed parameters were present. Method C: a weight to the interface loss was assigned such that its fraction is 50% of the total cost function, i.e. the PPI task was given more weight in this approach. Meaning that for Eq. (1) we have $\alpha = \beta + \gamma + \delta + \varepsilon$ and $\beta = \gamma = \delta = \varepsilon$.

### Model settings

In line with Xu et al.^17^, the batch size was set to 4 proteins, initial weights were set by the glorot uniform initializer, and the Adam optimizer is used during training^54^. The learning rate is divided by two when validation performance decreases, as measured by the area under the receiver operator characteristics curve (AUC ROC) of the PPI interface prediction.

We evaluated the early stopping criteria to avoid overfitting as defined for the OPUS-TASS model^17^ by training several models for 50 epochs. Xu et al. defined the stopping criteria to converge when for the fourth time the AUC ROC score on the validation set is lower than the previous score. Training the models further after reaching these stopping criteria did not show improvements in model performance. We therefore decided to use the same stopping criteria.

The single-task learner, called the IF model, is only trained on the PPI interface prediction task. This model was not able to identify interface residues when using an initial learning rate of 1e−3, the initial learning rate used to train the OPUS-TASS model. After hyperparameter tuning, the initial learning rate was set to 2.5e−4. Additionally, a weight was applied to the true interface residues in the loss calculation in order to adjust for the class imbalance of the PPI interface prediction. This weight was set to the ratio of non-interface residues over interface residues in the training and validation set. Therefore, this weight was set to 6.37.

Training and evaluation of the model is performed on one node containing a Titan X GPU. Models converge after approximately 10 epochs. On the PPI_extendedSFD dataset the duration of one epoch is approximately one hour. The validation performance of the model on all the prediction tasks together with training performances were collected with TensorBoard^53^.

### Evaluation

In order to measure the performance of the prediction models, the datasets were split into a training (80%), validation (10%), and test (10%) set (see Supplementary Fig. 1). We use the validation set for studying different multi-task learning models, model selection, and studying the partially annotated datasets. The test set is only used to confirm the performance of the best models.

We compare the performance of the single-task IF model to different multi-task models. The multi-task models contain different combinations of the related learning tasks as prediction tasks next to the interface prediction. After the models have seen all training sequences in one epoch, models are evaluated. We proceed training until the early stopping criteria, which is only based on the PPI interface prediction performance, is reached. Thereafter, the model outputs the highest reached AUC ROC score for the PPI interface prediction, as well as the corresponding scores of the related tasks, on the validation set.

We use different performance measures for the different prediction tasks. The performance of the main task on which we focus in this paper—PPI interface prediction—is determined by the AUC ROC. The ROC curve presents the relation between sensitivity and specificity at different classification thresholds. The area under this curve summarises the curve and represents the probability that the model yield a higher value for a residue that is in the interface than for a residue not in the interface^55^. To allow future comparison with (novel) PPI interface prediction methods, the accuracy, precision, recall, specificity, Matthews correlation coefficient (MCC), and F1-score are evaluated are provided in Supplementary Table 1. The secondary structure prediction in 3 classes and 8 classes, and the buried prediction performance is measured by the accuracy (ACC). Accuracy presents the probability of correctly predicting the class label. Compared to the AUC ROC the classification threshold is fixed at 0.5^56^.

The absolute solvent accessibility performance is measured by the Pearson correlation coefficient (PCC), which is a normalised measure of the covariance in the range between − 1 and 1^57^.

All models are trained four times, after which the mean performance and standard deviation on the validation set is determined. The different models are compared based on the PPI AUC ROC scores. A one-sided test of significance is performed on the difference of the two independent AUC ROC scores^58^, available from http://vassarstats.net/roc_comp.html.

We performed an error analysis on the individual proteins in the test set, to investigate the relation between small interfaces and IF performance scores. Furthermore, we test the relation between low IF prediction scores and the other structural feature task prediction scores, by performing a linear regression using the scipy.stats module (version: 1.3.1, see https://docs.scipy.org/doc/scipy/reference/stats.html).

## Results

In order to test whether PPI interface prediction could be improved when formulated as a multi-task problem, we generated two datasets with structural and PPI annotations: (1) a PPI dataset that contains both structural and PPI annotations for all proteins and (2) a PPI_extendedSFD dataset that contains structural annotations for all proteins and PPI annotations for only a third of the data. Hence, the PPI dataset (3551 proteins) is a subset of the PPI_extendedSFD dataset. The PPI_extendedSFD dataset (11,007 proteins) is augmented by the remaining protein structures in the OPUS-TASS dataset for which structural information—but not PPI annotations—are available, see also Table 1).

**Table 1:** The number of proteins in the training, validation and test sets for the PPI and PPI_extendedSFD datasets.

Structural information	PPI dataset		PPI_extendedSFD	
	SF + PPI	Only SF	SF + PPI	Only SF
Training set	2842	0	2842	5961
Validation set	353	0	353	749
Test set	356	0	356	746

The PPI dataset only contains proteins for which both structural features and PPI interface annotations are available. The PPI_extendedSFD dataset contains additional proteins for which only structural features are available.

### Tuning the multi-task model

We adjusted the multi-task model setup of Xu et al.^17^ to allow masking of unannotated labels during the training process and added a performance measure for the absolute solvent accessibility prediction task. In addition, the model was extended to identify buried and PPI interface residues. These tasks were implemented in the same way as the existing secondary structure classification tasks.

The initial learning rate was tuned on the PPI dataset by training the IF model on the training set and validating the model on the validation set. We tuned this parameter by considering the values 1e−3, 5e−4, 2.5e−4, 1e−4, 7.5e−4, 5e−5 and 1e−5. Model performances were measured by AUC ROC, area under the precision-recall curve (AUC PR), and accuracy. A stable optimum is reached by training the model on the learning rate of 2.5e−4 (see Supplementary Fig. 3). We evaluated the performance of the model on these learning rates, for the related tasks by training the best performing multi-task model presented by Xu et al.^17^. The results show, in line with the PPI interface performance score, a stable optimum for a learning rate of 2.5e−4 (see Supplementary Fig. 4).

### Performances of different multi-task learning models

We used the prediction tasks S3, S8, SA, and BU as possible related learning tasks to the PPI interface prediction task. Several models were trained on different combinations of these tasks. Every model was trained four times on both the PPI and PPI_extendedSFD dataset seperately after which the mean AUC ROC and AUC PR scores and their standard deviation was determined. Results, on the validation sets, are shown in Table 2. Results of the other considered performance measures are shown in Supplementary Table 1. The multi-task learning strategy significantly (P < 1e−3 for all models) outperforms the single task learner (AUC ROC: 73.17±0.36) on both the PPI and the PPI_extendedSFD dataset. The ‘IFBUS3SA’ model, trained on the PPI_extendedSFD dataset using interface (IF), secondary structure in three classes (S3), buried (BU) and solvent accessibility (SA) as prediction tasks reaches the highest AUC ROC (76.32±0.23). This multi-task model significantly outperforms the single-task model (P < 1e−6) and the IFBU model on the PPI dataset (P < 1e−3). Additionally, it outperforms the IFBU model on the PPI_extendedSFD dataset and the IFBUSA model on the PPI dataset significantly (P < 0.01). Including the more specific secondary structure classification task S8 instead of S3, or both S3 and S8, did not show further improvement.

**Table 2:** Comparison of the PPI interface performance of the single-task model against different multi-task models.

	PPI dataset		PPI_extendedSFD dataset	
	AUC ROC	AUC PR	AUC ROC	AUC PR
IF	73.17 ± 0.36	31.71 ± 1.01	73.17 ± 0.36	31.71 ± 1.01
IFBU	74.85 ± 0.19	34.37 ± 0.32	75.15 ± 0.20	35.35 ± 0.17
IFBUSA	75.08 ± 0.24	35.62 ± 0.97	75.92 ± 0.21	36.65 ± 0.42
IFBUS3SA	75.73 ± 0.50	35.79 ± 1.44	**76.32 ± 0.23**	38.44 ± 0.92
IFBUS8SA	75.73 ± 0.31	36.39 ± 0.74	76.20 ± 0.24	37.95 ± 0.52
IFBUS3S8SA	75.73 ± 0.21	36.46 ± 1.13	76.06 ± 0.14	38.16 ± 0.93

The mean AUC ROC and AUC PR scores and the corresponding standard deviations, on the validation set after training the models four times, are shown. Performance is measured on the validation set of both the PPI dataset and the augmented PPI_extendedSFD dataset. The multi-task models outperform the single task model (73.17 ± 0.36 AUC ROC) significantly on both dataset (P < 0.001). The overall highest AUC ROC score (76.32 ± 0.23), shown in bold, is reached when including buried residues, secondary structure in three classes and absolute solvent accessibility as related prediction tasks in addition to the PPI interface prediction on the PPI_extendedSFD dataset.

The PPI interface prediction performances expressed in AUC PR scores (see Table 2) follow similar trends to the AUC ROC scores. Further analysis showed that single-task learners of the related tasks optimised for that specific task reaches similar prediction performances as the multi-task learners optimised for PPI interface prediction (see Supplementary Fig. 5). PPI interface prediction AUC ROC scores attained by additional models trained on more combinations of the related tasks can be found in Supplementary Fig. 6.

We tried to improve the PPI interface predictions by including the torsion angle prediction as additional related learning task. We included the phi-angle and psi-angle prediction in all models described in Table 2. Performance was measured by the mean absolute error. However, after adding these tasks no significant improvement was shown compared to the best presented model (IFBUS3SA), see also Supplementary Table 2).

**Figure 3:**
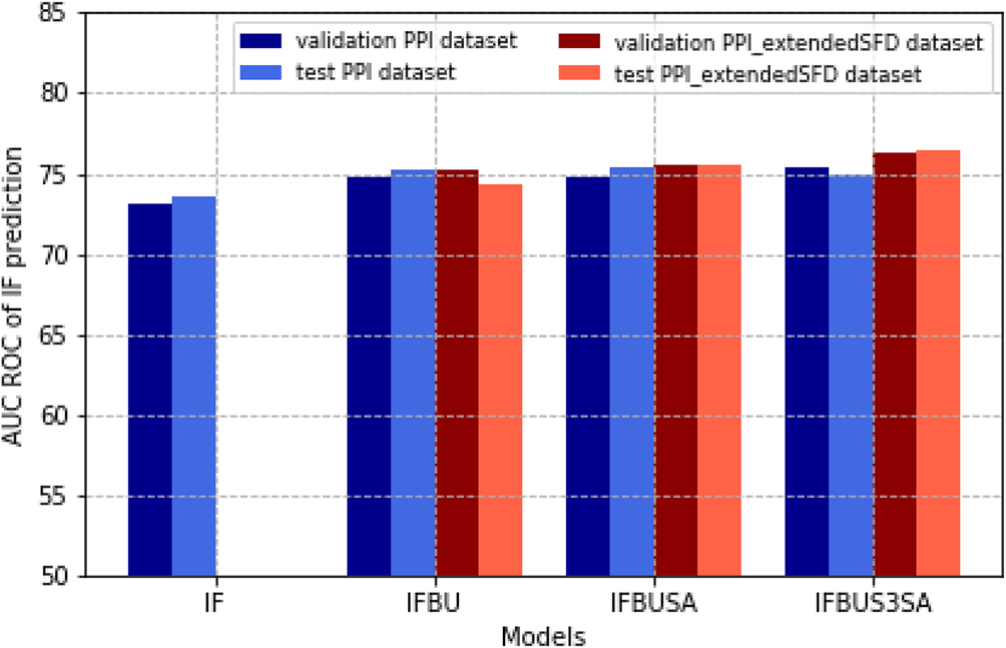
Comparison of the single-task model and multi-task models based on the AUC ROC scores of the PPI interface prediction on the validation and an independent test set. Performances are shown for the validation (dark blue) and test (blue) set for models trained on the PPI dataset, and the validation (dark red) and test (red) set for models trained on the PPI_extendedSFD dataset. All models are trained once on the training set. Similar performances are shown for the validation and test set. The multi-task models outperform the single-task model.

We tested our models on the independent test set. Results are shown in Fig. 3 and illustrate the similar performances of the PPI interface prediction expressed in AUC ROC scores. Supplementary Table 3) shows additional performance measures for both the test and validation sets. These results further support the conclusion that the multi-task learners outperform the single-task learner.

We investigated whether model performances could be improved by tuning the weights of the loss of the individual tasks in the total cost function using three different methods. Method A, for which the results are described above, weighs all tasks equally. Method B halves the weights of strongly related learning tasks (see “Methods” for further details). Method C keeps the contribution of the PPI interface prediction loss constant as 50% of the sum of the weights over all prediction tasks, this puts a much strong weight on the IF task. Both methods were tested during training of the IFBUS3SA, IFBUS8SA and IFBUS3S8SA models (see Supplementary Fig. 7A). Method B was also compared to models including only one of the similar prediction tasks (see Supplementary Fig. 7B). The results do not suggest an increase in model performance. Hence, the model does not appear to be very sensitive to the weights of the cost function.

### Partially annotated dataset

When comparing the model performances with and without data extension (in Table 2 and Fig. 3) we can observe a subtle increase in performance for the training datasets extended with structural property information, but without additional PPI interface annotations. These results suggest that the interface prediction benefits not only from the multi-task learning strategy by annotating the protein sequences in the PPI dataset by the related task information, but also from training on additional data of the related tasks only, as provided by the partially annotated dataset.

**Figure 4:**
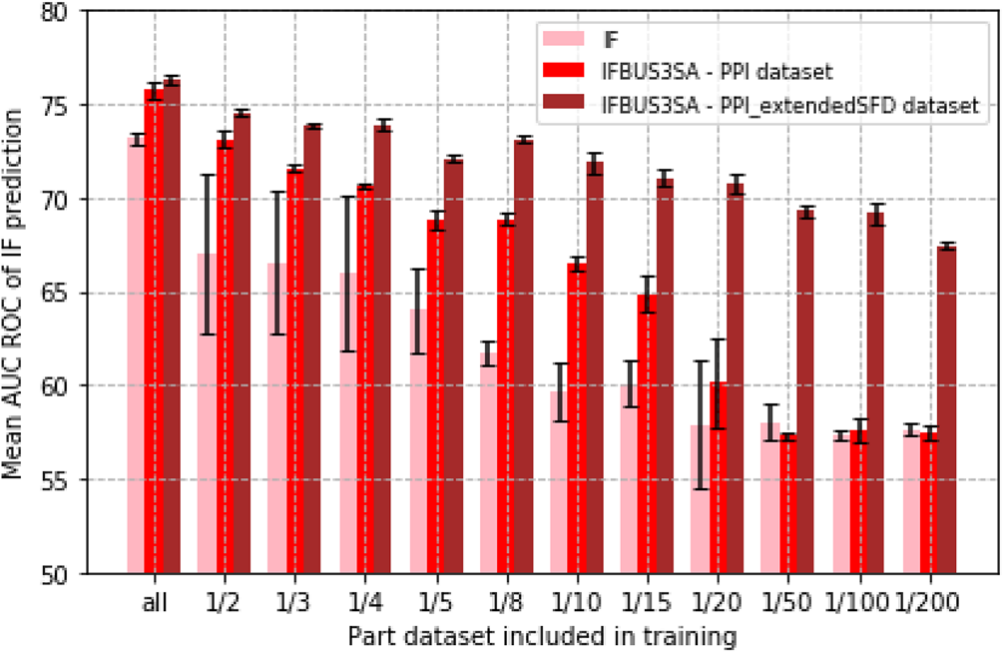
The importance of the multi-task setup and the data extension when training a PPI interface prediction model trained on limited data. The single-task model IF (pink) and the multi-task model IFBUS3SA (red and brown) are compared. The IF model and the IFBUS3SA model indicated in red are trained on a part of the PPI dataset. Differences in performance between the pink and red bars therefore presents the benefit of the multi-task learning strategy. The IFBUS3SA model in brown is trained on the PPI_extendedSFD dataset in which only a part of the PPI interface information is considered. All the brown bars are thus trained on the same number of sequences for which the related task information is available. Differences in performance between red and brown bars indicate the benefit of training the model on the augmented PPI_extendedSFD dataset. Model performance is shown by the mean AUC ROC (bars) and standard deviation (whiskers) of the PPI interface prediction on the total validation set.

To further investigate these results we decreased the PPI interface annotations in the datasets. We evaluated the single-task learner and the best performing model IFBUS3SA on both datasets in which only a part of the data is considered, see Fig. 4. We trained the single-task model and the IFBUS3SA model on a part of the PPI dataset. Next, we trained the IFBUS3SA model on the PPI_extendedSFD dataset for which we included only a part of the PPI annotations. At each data decreasing step, the three models are trained on the same PPI interface information and all models are evaluated on the total validation set. Model performance is measured by AUC ROC for the PPI IF prediction.

Figure 4 shows that, as expected, less training data generally leads to a worse performance for all strategies. For very small training datasets (e.g. one-twentieth of the total data) the multi-task learning (red bars), without extending the data, does not outperform the single task strategy (pink bars) significantly (Fig. 4). This is probably also due to the lack of sufficient information to train on.

Figure 4 also shows that the smaller the training datasets the larger the difference in performance between the single-task strategy (pink bars) and multi-task strategy with data extension (brown bars). Hence, the performance of the multi-task model improves strongly when the training dataset is augmented with proteins only containing structural annotations, but for which the PPI annotations are missing, i.e. when it is trained on the PPI_extendedSFD dataset (brown compared to red). Thus, information captured by the related learning tasks improves PPI interface prediction even if PPI interface annotations are not available for the majority of the proteins in the training set. Likewise, the data extension becomes more important if annotated PPI interface data is very scarce. Equal performances are reached for the IF model trained on the entire available PPI interface information (pink bar labelled ‘all’) and the IFBUS3SA model trained on the PPI_extendedSFD dataset including only one eighth of the available PPI interface information (brown bar labelled ‘1/8’).

To confirm these results we also tested the models including all, 1/2, 1/, 1/20, and 1/200 of the data on an independent test set. Results are shown in Supplementary Fig. 8 and are in line with the results described above.

### Error analysis

**Figure 5:**
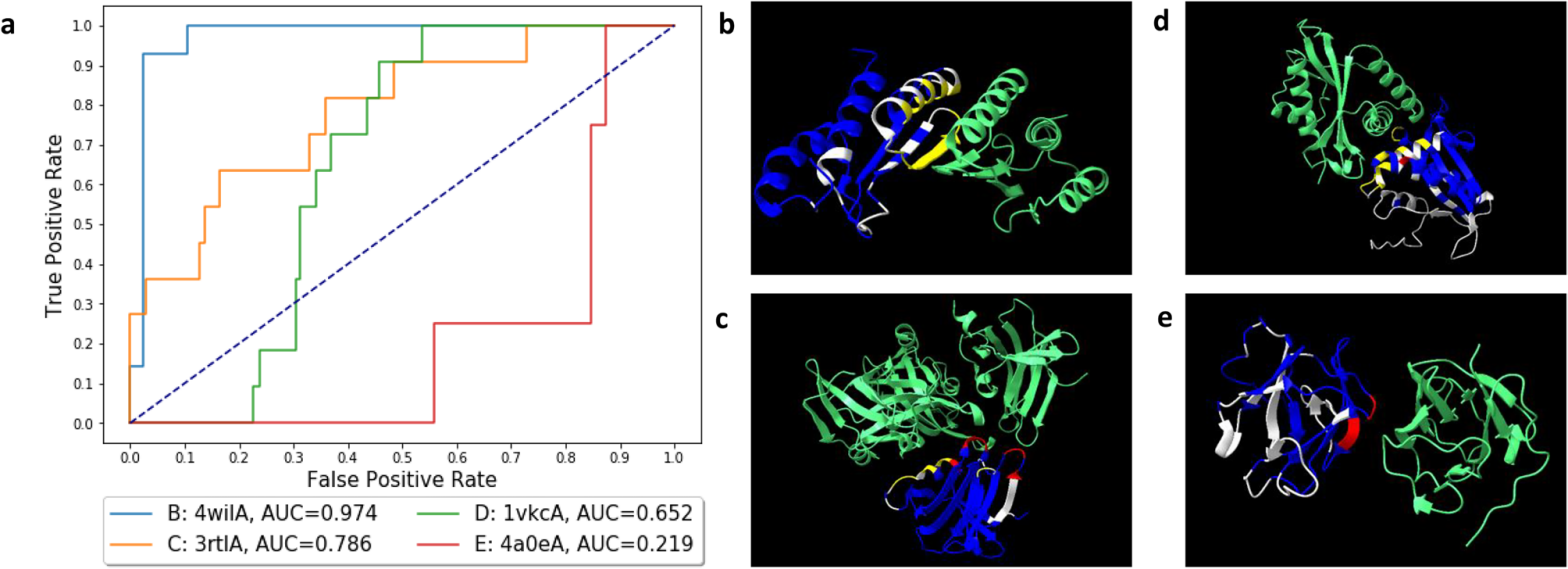
Analysis of four proteins in the test set after training the multi-task model IFBUS3SA on the PPI_extendedSFD dataset. (**a**) The receiver operator characteristics (ROC) curve of the four proteins (4wilA in blue, 3rtlA in orange, 1vkcA in green, and 4a0eA in red) with their corresponding area under the curve score (AUC ROC). (**b**–**e**) Visualised protein structures corresponding to the proteins in the ROC curve. The protein chains that contain the predicted interface are indicated in dark blue, the binding partners are indicated in green. Correctly predicted residues are colored yellow, false positive residues white and false negative residues red. (**b**) Protein structure of the protein 4wilA, corresponding to light blue line in the ROC curve. (**c**) Protein structure of the protein 3rtlA, corresponding to the orange line in the ROC curve. (**d**) Protein structure of the protein 1vkcA, corresponding to the green line in the ROC curve. (**e**) Protein structure of the protein 4a0eA, corresponding to the red line in the ROC curve.

We performed an error analysis on individual proteins in the test set after training the multi-task model IFBUS3SA to gain biological insight in the predictions generated by the models. Figure 5 shows receiver operator characteristics curve for four single exemplary proteins. Target and predicted residues are shown using structure viewer UCSF ChimeraX^59^ (see Fig. 5).

The proteins with high AUC ROC values show many correctly predicted interface residues (indicated in yellow in Fig. 5b–d). False positive residues, which are residues predicted as interface residues but not indicated as such in the gold standard (indicated in white in Fig. 5b–e), are typically closely located to the actual interface for those (see Fig. 5b). Proteins corresponding to lower AUC ROC values show some false negatives, which are interface residues that are not predicted as interface residues by the model (indicated in red in Fig. 5c–e), and many false positives. Note that some false positives occur in localised regions of the structure, possibly indicating a true PPI interface region which is not annotated in the dataset. For example, in Fig. 5b the false positives located on the $\alpha$-helices, actually form a secondary interface in the tetrameric structure. For one protein, with a very small interface, no correctly predicted residues were observed (see Fig. 5d).

**Figure 6:**
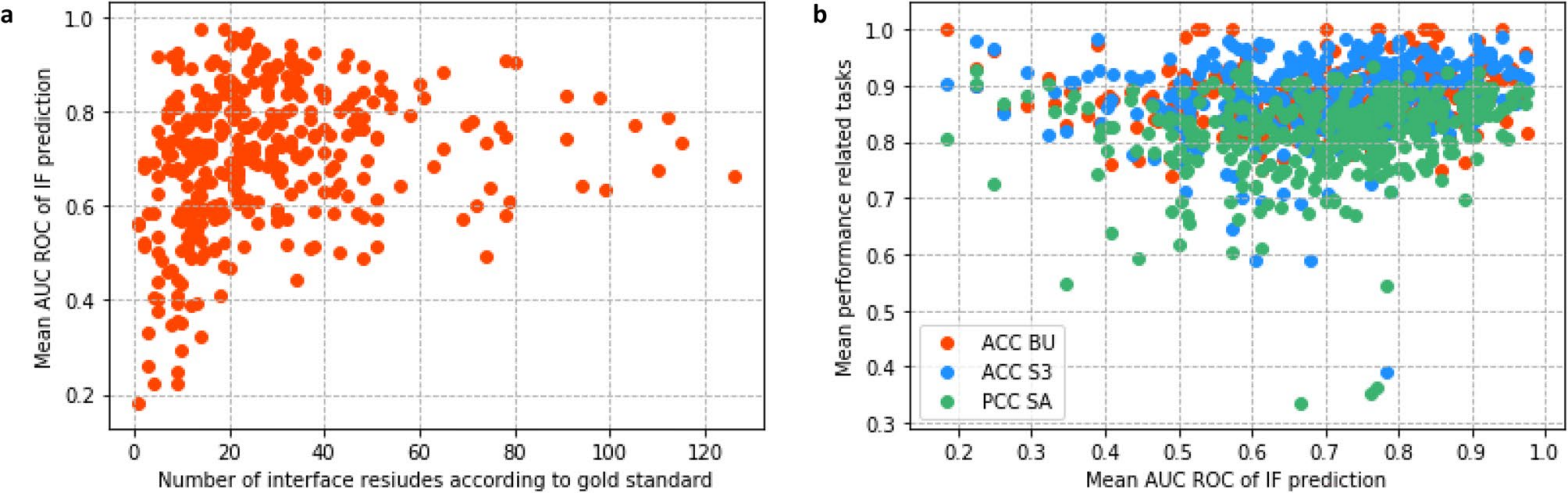
Error analysis on the individual proteins in the test set after training the IFBUS3SA model on the PPI_extendedSFD dataset. (**a**) The mean AUC ROC of the interface prediction is plotted against the number of interface residues per protein. Low AUC ROC scores (< 0.4) are only observed when the interface region of the protein is small (< 20 residues). (**b**) The mean accuracy of the predicted structural features (BU, S3 and SA), and the mean Pearson correlation coefficient of the absolute solvent accessibility are plotted against the mean AUC ROC score of the PPI interface prediction per protein. Linear regression was performed resulting in an R^2^ of 0.010 (BU), 0.016 (S3) and 0.031 (SA).

To analyse the error trends in more detail, we trained four different multi-task models, using different training rounds, and determined the mean performance per protein in the test set. Figure 6a shows that proteins with low (< 0.4) AUC ROC values for the PPI interface prediction are all proteins containing a small annotated interface region. However, proteins containing small interfaces do not necessarily result in low prediction scores. Moreover, we studied the relation between the PPI interface prediction and the related task predictions. This was done in order to test whether proteins corresponding to low AUC ROC scores are, in general, proteins for which structural features are hard to predict. The ${R}^2$ was determined and resulted in 0.010 for the buried residues, 0.016 for the secondary structure in three classes and 0.031 for the absolute solvent accessibility. Hence, no considerable correlation was found between the AUC ROC value of the PPI interface prediction and the related learning task for the IFBUS3SA model (see Fig. 6b). The same conclusion was drawn after performing this analysis on the IFBUS3SA model trained on only one tenth of the PPI interface information (see Supplementary Fig. 9).

## Discussion

Predicting the protein–protein interaction interface from sequence is a difficult task and annotations of interface residues are scarce. Here, we show how to overcome the problem of the limited size of the datasets by training a deep neural network predicting PPI interface residues using a multi-task learning strategy on a partially annotated dataset. All our multi-task models outperform the single-task model significantly (P < 0.001) on the PPI and the PPI_extendedSFD datasets. The single task model achieves AUC ROC: 73.2% ± 0.4, while the best performing multi-task model reaches 76.3% ± 0.2; this latter model includes as related prediction tasks the identification of buried residues, secondary structure, and absolute solvent accessibility, in addition to predicting the PPI interface. Performances on the independent test set are in line with the results on the validation set. Hence the representations learned by the related structural annotation tasks can indeed help the model with the task of classifying PPI interface residues.

We show that the added benefit of the multi-task setup can be further increased by adding annotations for only the related tasks: the PPI interface prediction drastically improved when we extended the limited PPI training dataset with additional samples (proteins) for which only related structural annotations were available. Moreover, the multi-task setup becomes even more important when the training set is reduced. To highlight the strength of this, we removed all but one-eight of the PPI interface information from the extended dataset. In this scenario the multi-task model still achieves similar performance scores as the single-task learner when trained on all PPI interface information. Hence, we show that formulating a predication task as a multi-task learning problem can be immensely powerful for protein structural (or functional) prediction tasks for which only a small set of annotated training data is available.

We hypothesise that the shared model representation allows to learn fundamental properties of the protein structure. Learning related tasks—such as which amino acids are exposed to the surface—will make the learned representation more relevant. In particular it is to be expected that information on surface accessibility will be crucial for the decision if a residue is an interface residue or not. Moreover, there are notable difference in the between the amino acid composition of surface, interface and core residues^60^. Klausen et al.^15^ and Xu et al.^17^ already showed co-learning of secondary structure and surface accessibility can make the learning models more accurate. Here we show two additional points (1) functional annotations on protein structure also benefit from this shared representation and (2) the multi-task setting become especially powerful if for one of the tasks a very limited amount of data is available.

It is important to mention that in the current structural datasets, it is likely that many true protein–protein interaction (PPI) interface residues are not annotated as such, simply because no PDB structures of the relevant bound states are available. Hence, some true protein–protein binding interfaces will be missing in any structural dataset used for training and performance evaluation of any PPI interface prediction method.

The scope of this study is to provide a proof of principle for the use of multi-task learning to improve prediction performance for protein structure tasks with scarce functional annotations, such as PPI interfaces. Our learning model is comparable to the multi-task model used in OPUS-TASS^17^, with the additional ability to train on partially annotated data. We did not perform extensive tuning of the architecture to obtain the highest possible PPI interface prediction accuracies. Nevertheless, our resulting AUC ROC scores are comparable to published state of the art methods for PPI interface prediction. Note that the performance of these different methods cannot be compared directly as different test sets were used. We will, however, include a discussion of the measured performance of these models to provide a background against which to better interpret our results, and to compare which features were included, and how these were used in the prediction model. A recent overview of state of the art PPI predictors is provided by Zhang & Kurgan^9^, which we recently extended with a comparison with some of our own methods^23^. These comparisons were all done on their ZK448 benchmark dataset^14^ using several metrics, below we list their AUC-ROC scores (unless noted otherwise). SSWRF by Wei et al.^30^ achieved an AUC-ROC of 68.7%; it outperformed the state-of-art methods in 2016. Similarly to our multi-task model, this method used information of the PSSM and the solvent accessibility. In SSWRF these are both used as input feature, whereas in our model the latter is used as related learning task. Their third input feature, the averaged cumulative hydropathy, was not considered for our method. SeRenDIP by Hou et al.^7,31^ later achieved an AUC ROC score of 70.7% on a dataset of both homomeric and heteromeric protein complexes (HHC). SeRenDIP was not compared to SSWRF directly, but on the HHC test set did outperform older PPI prediction models SPPIDER^61^ and PSIVER^62^. SeRenDIP uses conservation, solvent accessibility, and secondary structure as input features to generate the PPI interface predictions. Furthermore, SeRenDIP includes backbone dynamics and sequence length, which are not used in our current multi-task model. SCRIBER achieves a AUC ROC of 71.5%, outperforming among others SPPIDER (51.7%), PSIVER (58.1%) and SSWRF on their ZK448 test set^9^. SCRIBER uses evolutionary conservation, relative solvent accessibility, and secondary structure features as input features. Furthermore, SCRIBER uses amino-acid physicochemical properties as input (charge, hydrophobicity, polarity, aliphaticity, aromaticity, acidity and size), as well as relative amino acid interface propensity and annotations of intrinsically disordered regions. Note that additional features, such as those used in other methods, may also be included in our multi-task model, either as input features or as related learning task. For example, we have previously shown that including sequence length and backbone flexibility as input features improves prediction of PPI^7^ and epitope regions^13^. In addition, structure based features, such as (predicted) residue contacts, or other (predicted) 3D features could be added as input, or as related task. Recent advances in 3D structure prediction^2^, suggests that this may be a likely way to further improve PPI interface prediction. However, the architecture of the model would have to be adapted. Several studies presented models to predict the interaction between proteins and other molecules like peptides, small molecules, and nucleic acids^9,23,63^. These annotations could potentially be used as related tasks, and may further improve the model performance.

Although we did not perform extensive tuning of the model, we did investigate some parameters that are most likely to affect the multi-class learning. We implemented the multi-task learning strategy by the combined loss function which is used in all layers of the model (except the output layer). These layers are shared between all included tasks. We explored adjusting the weights of the different task, however, this did not result in any improvement of performance.

We furthermore tried to improve the PPI interface predictions by including torsion angle prediction, using the OPUS-TASS labels. However, no significant improvement was shown for the best presented model in this paper. In this work, we adjusted the initial learning rate, created our own additional output labels, and set weights for the combined loss function and the class imbalance for the PPI interface prediction. We hypothesize that similar PPI interface prediction performances could be achieved when simplifying the model architecture. Further studies should conclude if the performance could be improved even further when fine-tuning the model. Nevertheless, here we show the substantial benefit of the multi-task learning strategy on a partially annotated dataset to achieving accurate performances for the difficult PPI interface prediction task. Therefore, we hope that the multi-task setup and the data extension will be of significant value in other protein structural or functional prediction tasks in which the size of annotated training sets is (extremely) limited.

## Supplementary Information

Supplementary Information.

## Data Availability

The code is available at https://github.com/ibivu/multi-task-PPI. Note that all experimental protein structures used in this study have been deposited to the PDB^43^. The PDB accession codes, as well as the generated features and output labels used for training and validation is available at https://ibi.vu.nl/downloads/multi-task-PPI/.
