# Supplementary material for: Multi-task learning to leverage partially annotated data for PPI interface prediction
Source: Sci Rep. 2022 Jun 21;12:10487. doi: 10.1038/s41598-022-13951-2 (PMC9213449; doi:10.1038/s41598-022-13951-2)
Supplement: Supplementary file 1 — Supplementary Information. [file 41598_2022_13951_MOESM1_ESM.pdf]

Supplementary information for
Multi-task learning to leverage partially annotated data for PPI interface prediction

Henriette Capel[1], K. Anton Feenstra[1], Sanne Abeln[1,*]

[1] IBIVU – Center for Integrative Bioinformatics, Vrije Universiteit Amsterdam, Amsterdam 1081HV, The Netherlands

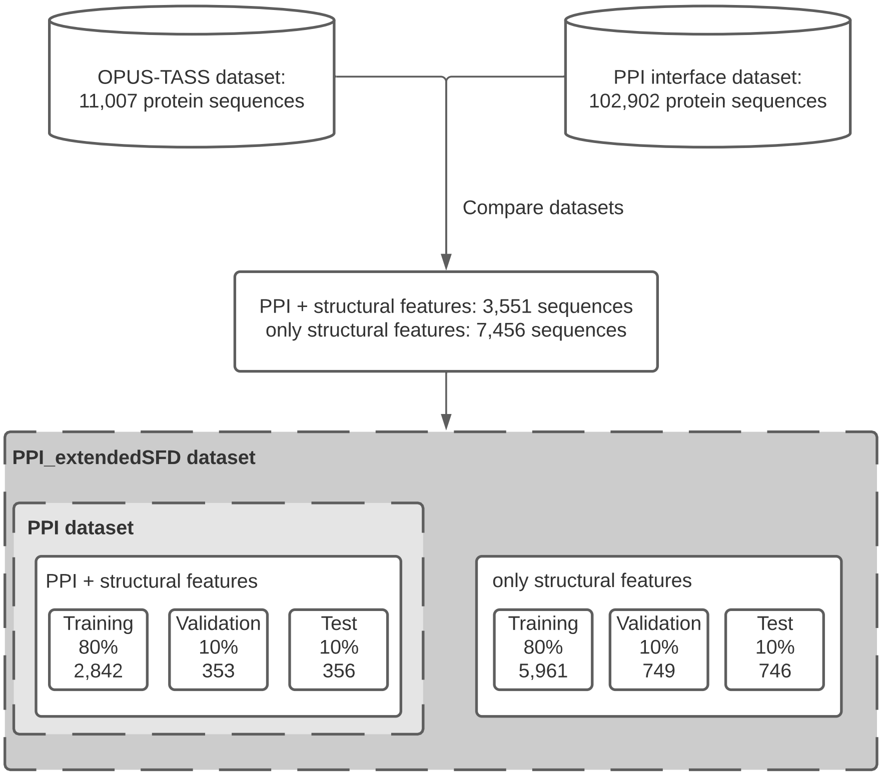

Supplementary Fig. 1. Construction of the PPI and PPI_extendedSFD dataset based on the combined training and validation dataset of OPUS-TASS. For one third of this dataset PPI interface annotations were available and stored in the PPI interface dataset. The PPI dataset consist of all proteins of the OPUS-TASS dataset for which PPI interface annotations are available. This dataset is a subset of the PPI_extendedSFD dataset which is augmented by the residual proteins of the OPUS-TASS dataset.

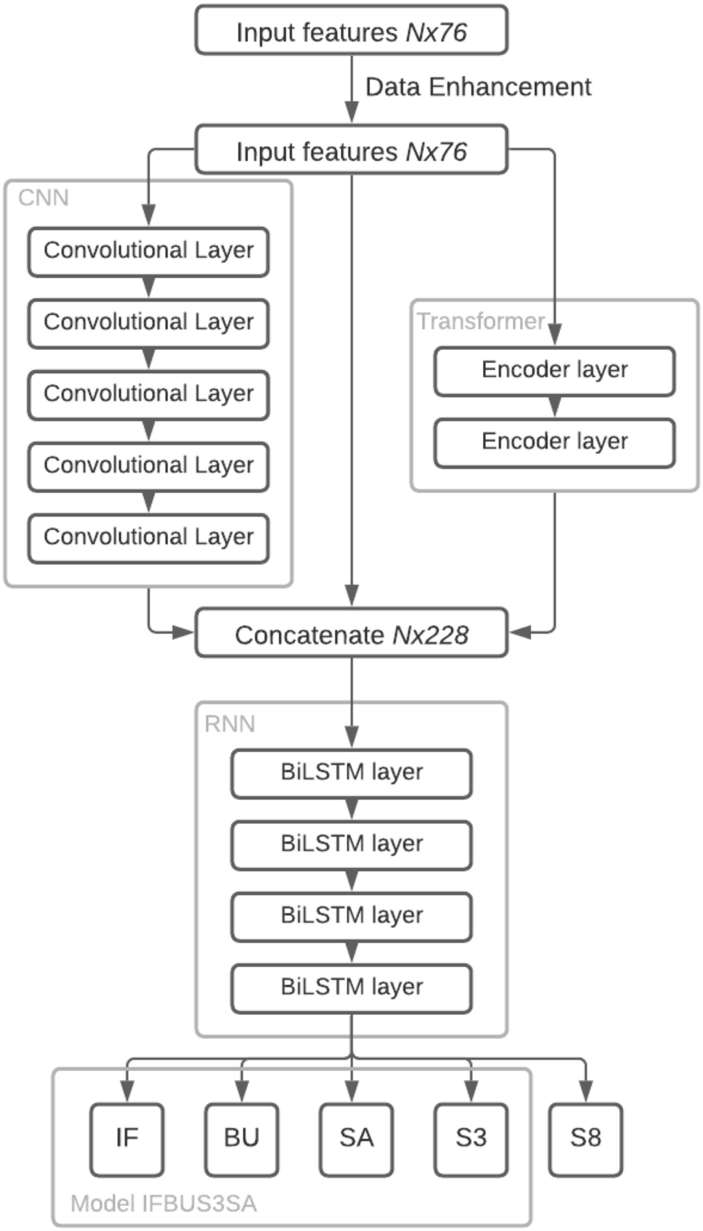

Supplementary Fig. 2. The architecture of the deep learning model based on the OPUS-TASS model of Xu et al. (2020). The input of the model is a matrix of size: length of the proten (N) by the 76 input features. The model consist of a convolutional neural network (CNN), a transformer, and a recurrent neural network (RNN), specifically a bidirectional long short-term memory (LSTM). The output of the model are the possible prediction tasks. The predictions for the different tasks are coupled via the network architecture, and their inclusion in the cost function.

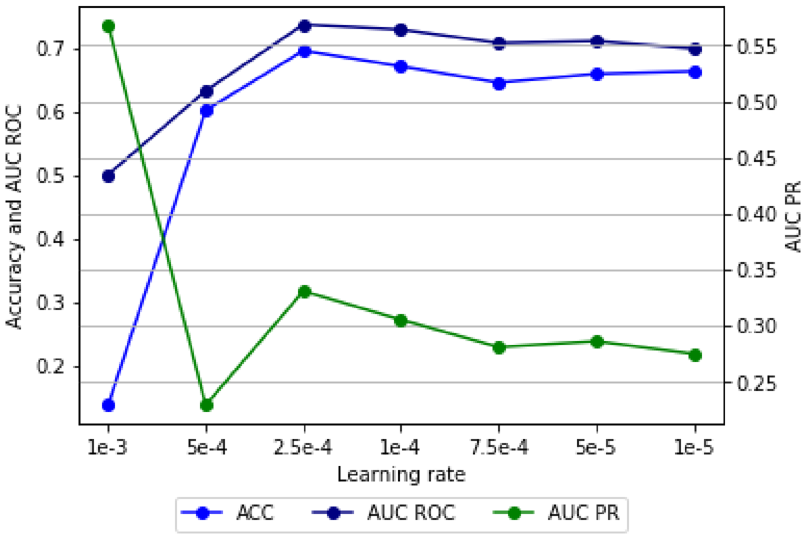

Supplementary Fig. 3. Model performance of the single task model on the PPI interface prediction task for different initial learning rates. The models are trained on the PPI dataset and performance is measured by the accuracy (ACC, in blue), area under the receiver operator characteristics curve (AUC ROC, in dark blue) and area under the precision-recall curve (AUC PR, in green) on the validation set. Highest prediction performances are shown for the initial learning rate 2.5e-4. Note that the highest AUC PR score at 1e-3 was only obtained when all residues where predicted to be non-interface residues.

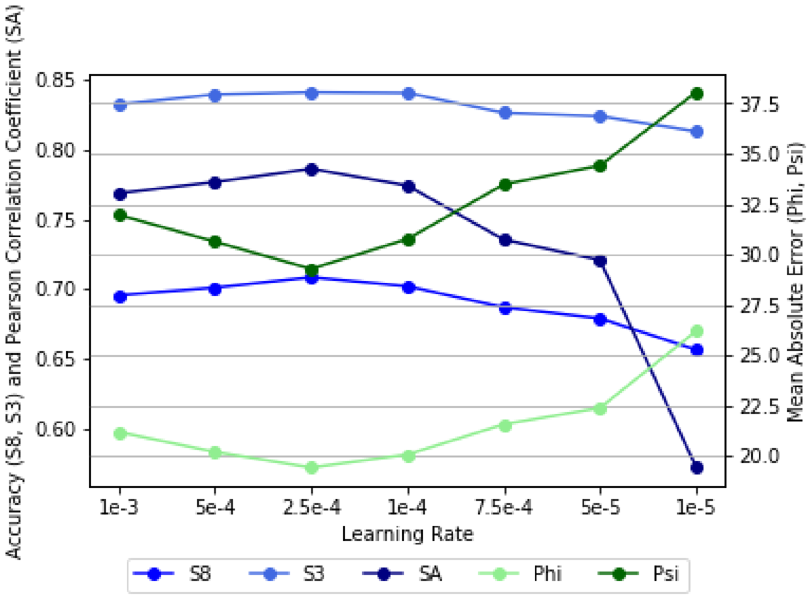

Supplementary Fig. 4. The model performance on the different prediction tasks (see legend) for different initial learning rates. The model used, is the best performing model (C4) in the work by Xu et al. (2020). The presented performances are accuracy of the secondary structure prediction in three (S3,light blue) and eight (S8, blue) classes, pearson correlation coefficient of the absolute solvent accessibility prediction (SA, dark blue), and the mean absolute error of the phi and psi angle (Phi and Psi, in light and dark green). The highest accuracy and pearson correlation coefficient, and the lowest mean absolute error for all tasks is reached by using the initial learning rate of 2.5e-4. This result is in line with the hyperparamter tuning result based on the PPI interface prediction.

Model	Dataset	accuracy	precision	recall	specificity	MCC	F1 score
IF	PPI	69.25 ± 1.31	25.26 ± 0.66	63.32 ± 1.90	70.18 ± 1.54	21.18 ± 0.63	36.10 ± 0.41
IFBU	PPI	71.96 ± 1.06	26.35 ± 1.41	62.34 ± 0.89	73.50 ± 1.21	23.63 ± 0.63	37.85 ± 0.45
IFBU	PPI_extendedSFD	70.07 ± 2.17	26.31 ± 1.14	65.27 ± 2.63	70.85 ± 2.54	22.95 ± 1.04	37.45 ± 0.68
IFBUSA	PPI	74.47 ± 1.72	28.89 ± 1.19	58.54 ± 3.18	77.13 ± 0.20	24.88 ± 0.75	38.55 ± 0.39
IFBUSA	PPI_extendedSFD	73.86 ± 3.62	29.08 ± 2.31	61.39 ± 5.67	75.85 ± 4.45	25.68 ± 1.55	39.30 ± 0.91
IFBUS3SA	PPI	70.07 ± 4.57	26.64 ± 2.14	66.11 ± 6.33	70.63 ± 5.42	23.40 ± 1.76	37.78 ± 1.13
IFBUS3SA	PPI_extendedSFD	74.68 ± 5.15	30.13 ± 3.86	60.25 ± 8.32	76.98 ± 6.34	26.45 ± 2.40	39.68 ± 1.40
IFBUS8SA	PPI	73.50 ± 4.20	28.69 ± 2.73	60.67 ± 6.99	75.50 ± 5.18	24.95 ± 1.71	38.68 ± 0.89
IFBUS8SA	PPI_extendedSFD	75.89 ± 1.37	30.38 ± 1.03	58.42 ± 2.79	78.68 ± 1.72	26.73 ± 0.74	39.95 ± 0.52
IFBUS3S8SA	PPI	72.97 ± 4.71	28.53 ± 2.94	62.07 ± 6.76	74.70 ± 5.63	25.03 ± 2.10	38.83 ± 1.26
IFBUS3S8SA	PPI_extendedSFD	74.50 ± 4.41	29.66 ± 3.04	60.01 ± 7.29	76.78 ± 5.41	25.98 ± 1.88	39.38 ± 1.04

Supplementary Table 1. Comparison of the PPI interface performance of the single-task model against different multi-task models. The accuracy, precision, recall, specificity, Mathews correlation coefficient (MCC), and F1 scores, and the corresponding standard deviations based on the four trained models, are shown. This table is an extension of Table 2. Performance is measured on the validation set of both the PPI dataset and the augmented PPI_extendedSFD dataset.

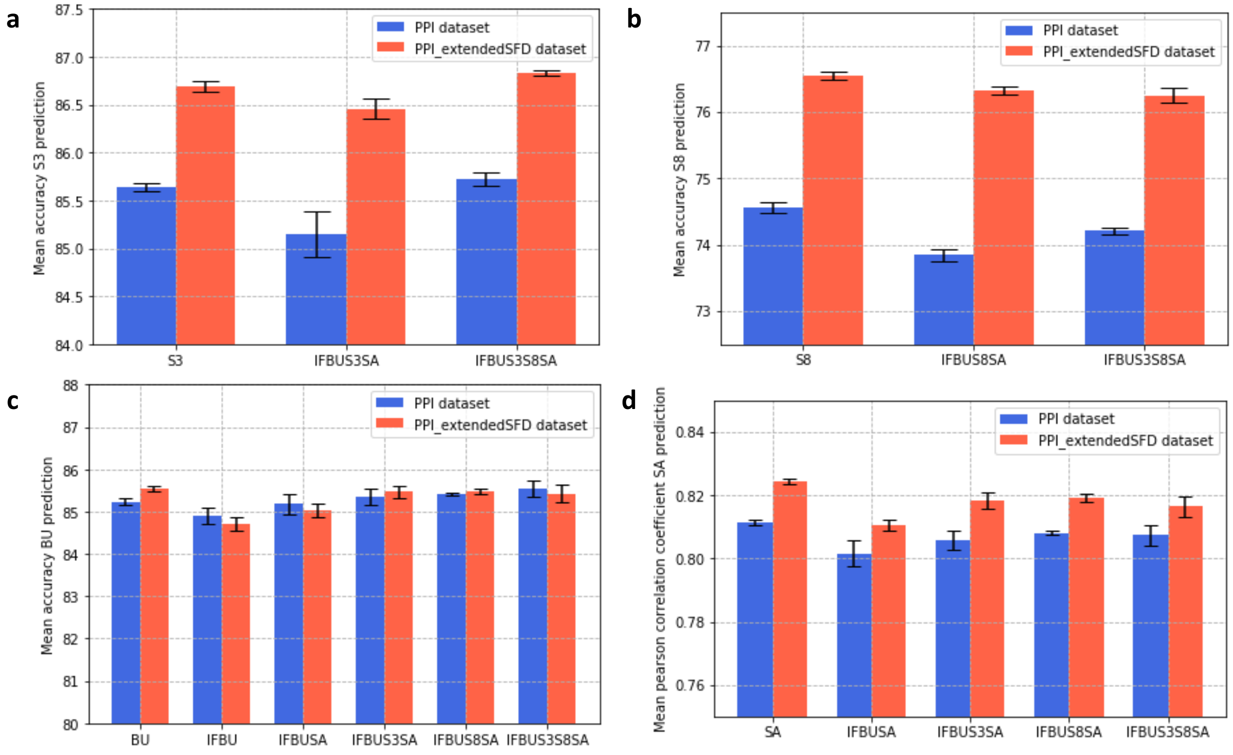

Supplementary Fig. 5. Model performances for the related prediction task of the single-task models and the multi-task models. Note that each single-task model is optimised by using its single-task performance measure in the stopping criteria and the multi-task models are optimised for the PPI interface prediction. All models are trained four times on both the PPI and PPI_extendedSFD dataset. Note that the extended dataset contains training labels for all tasks, except for the IF task which is only partially annotated. Mean performance and standard error are shown for the validation set. The multi-task models attain in most cases similar performance as the single-task models. (**a**) Model performances measured in accuracy of the secondary structure prediction in three classes. (**b**) Model performances measured in accuracy of the secondary structure prediction in eighth classes. (**c**) Model performances measured in accuracy of the buried residue prediction. (**d**) Model performances measured in pearson correlation coefficient of the absolute solvent accessibility prediction.

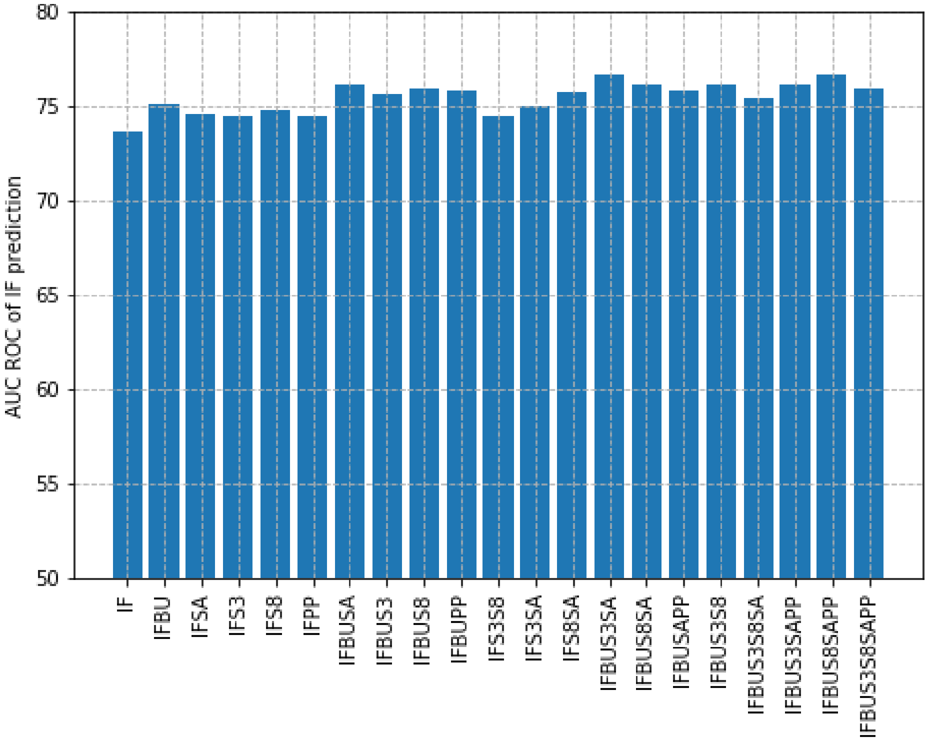

Supplementary Fig. 6. Comparison of the single-task model (IF) against multiple multi-task models. Model performance is measured by the area under the receiver operator characteristics curve (AUC ROC). All models are trained once and performance is shown for the validation set.

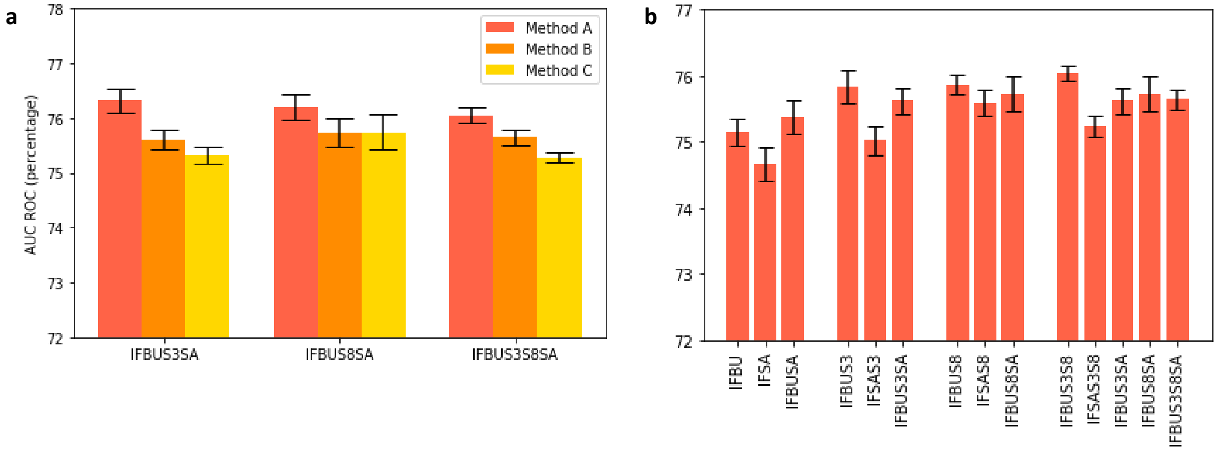

Supplementary Fig. 7. Increasing the weight of the fraction of the PPI interface prediction loss in the cost function. (**a**) Comparing the standard method in which the weights are set to 1.0 (Method A, red), against two different weighting methods. For Method B (orange) we halved the weights of pairs of similar prediction tasks, such both sum up to a weight equal to the other tasks. For Method C (yellow) we assigned a weight to the interface loss such that its fraction is 50% of the total cost function. The AUC ROC scores on the interface prediction tasks is shown for models IFBUS3SA, IFBUS8SA and IFBUS3S8SA. (**b**) AUC ROC scores on the interface prediction task are shown for models in which Method B is applied. Note that a weight is kept the same as in Method A if only one of the two correlated prediction task is present. The models are grouped based on the prediction tasks they included and are considered as similar. All performances are obtained on the validation set.

Model	Dataset	S8 (ACC)	S3 (ACC)	BU (ACC)	SA (PCC)	PHI (MAE)	PSI (MAE)	IF (AUC ROC)
IF	PPI							0.7364
IFPP	PPI					18.53	27.39	0.7486
IFPP	PPI_extendedSFD					17.94	26.17	0.7441
IFBUPP	PPI			0.8527<br>**+0.0037**		18.47	27.25	0.7550<br>**+0.0042**
IFBUPP	PPI_extendedSFD			0.8538<br>**+0.0066**		17.75	25.61	0.7582<br>**+0.0078**
IFBUSAPP	PPI			0.8548<br>**+0.0030**	0.8087<br>**+0.0070**	18.26	26.92	0.7515<br>+0.0006
IFBUSAPP	PPI_extendedSFD			0.8550<br>**+0.0046**	0.8206<br>**+0.0100**	17.65	25.38	0.7585<br>**-0.0030**
IFBUS3SAPP	PPI		0.8563<br>**+0.0050**	0.8532<br>-0.0003	0.8069<br>+0.0010	18.59	26.72	0.7601<br>+0.0028
IFBUS3SAPP	PPI_extendedSFD		0.8676<br>**+0.0030**	0.8549<br>+0.0002	0.8197<br>+0.0015	17.56	24.96	0.7617<br>**-0.0046**
IFBUS8SAPP	PPI	0.7449<br>+0.0064		0.8549<br>+0.0007	0.8092<br>**+0.0010**	18.09	26.41	0.7586<br>-0.0006
IFBUS8SAPP	PPI_extendedSFD	0.7667<br>+0.0034		0.8556<br>+0.0008	0.8208<br>**+0.0015**	17.28	24.31	0.7664<br>**+0.0049**
IFBUS3S8SAPP	PPI	0.7470<br>+0.0049	0.8587<br>+0.0015	0.8587<br>**+0.0034**	0.8091<br>+0.0017	18.19	26.23	0.7515<br>**-0.0060**
IFBUS3S8SAPP	PPI_extendedSFD	0.7658<br>**+0.0033**	0.8690<br>+0.0007	0.8560<br>+0.0017	0.8189<br>+0.0023	17.46	24.51	0.7596<br>**+0.0052**

Supplementary Table 2. Comparison of the multi-task models including the torsion angle predictions (PP) as related prediction tasks against models without this prediction. Performances for the phi- and psi- angles are measured by the mean absolute error (MAE) and are shown for the validation set after training the models once. Performance for S3, S8, and BU are measured in accuracy (ACC), performance of SA is measured in the Pearson correlation coefficient (PCC), and performance of IF is measured in the area under the ROC curve. The plus and minus signs indicate the increase or decrease of the models including the torsion angles compared to the model without this prediction task. Values in bold indicate that they lie outside the standard error region.

Model	Dataset	accuracy	precision	recall	specificity	MCC	F1 score
IF	PPI validation	68.38	24.86	64.56	68.99	20.82	35.90
IF	PPI test	67.28	25.29	67.62	67.22	21.31	36.81
IFBU	PPI validation	67.14	24.88	69.17	66.81	21.58	36.60
IFBU	PPI test	66.23	25.40	72.07	65.28	22.17	37.56
IFBU	PPI_extended validation	69.48	26.03	66.52	69.95	22.81	37.42
IFBU	PPI_extended test	68.05	25.61	66.50	68.31	21.60	36.98
IFBUSA	PPI validation	74.32	28.73	58.96	76.76	24.93	38.64
IFBUSA	PPI test	74.29	29.67	60.14	76.61	25.81	39.73
IFBUSA	PPI_extended validation	75.93	30.09	57.02	78.94	26.12	39.39
IFBUSA	PPI_extended test	75.47	30.21	56.50	78.58	25.65	39.37
IFBUS3SA	PPI validation	75.88	30.08	57.29	78.84	26.16	39.43
IFBUS3SA	PPI test	74.16	29.28	58.89	76.67	25.08	39.12
IFBUS3SA	PPI_extended validation	78.41	32.49	53.27	82.41	27.87	40.37
IFBUS3SA	PPI_extended test	76.38	32.74	54.10	81.76	27.90	40.79

Supplementary Table 3. Comparison of the single-task model and multi-task models on the accuracy, precision, recall, specificity, Matthews correlation coefficient (MCC), and F1-score of the PPI interface prediction on the validation and an independent test set. This table is an extension of Figure 3. Performances are shown for the validation and test set for models trained on the PPI dataset, and the validation and test set for models trained on the PPI_extendedSFD dataset. All models are trained once on the training set. In general, similar performances are shown for the validation and test set. For all performance measures except recall, the multi-task models outperform the single-task model.

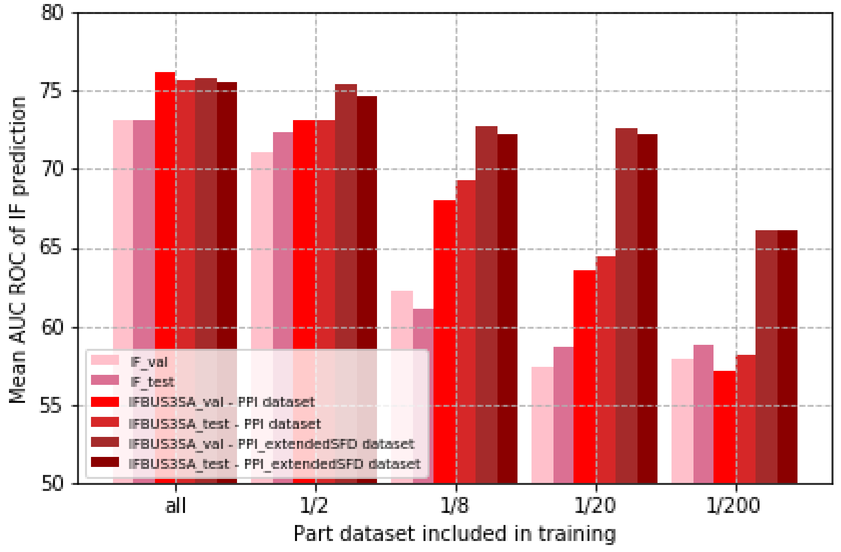

Supplementary Fig. 8. The importance of the multi-task setup and the data extension when training a PPI interface prediction model on limited data. Model performance is shown by the AUC ROC on the PPI interface prediction on the total validation and test set. Results on the test set are in line with the results on the validation set. The IF model and the IFBUS3SA model indicated in red are trained on a part of the PPI dataset. Differences in performance between the pink and red bars therefore presents the benefit of the multi-task learning strategy. The IFBUS3SA model in brown is trained on the PPI_extendedSFD dataset in which only a part of the PPI interface information is considered. All the brown bars are thus trained on the same number of sequences for which the related task information is available. Differences in performance between red and brown bars indicate the benefit of training the model on the augmented PPI_extendedSFD dataset.

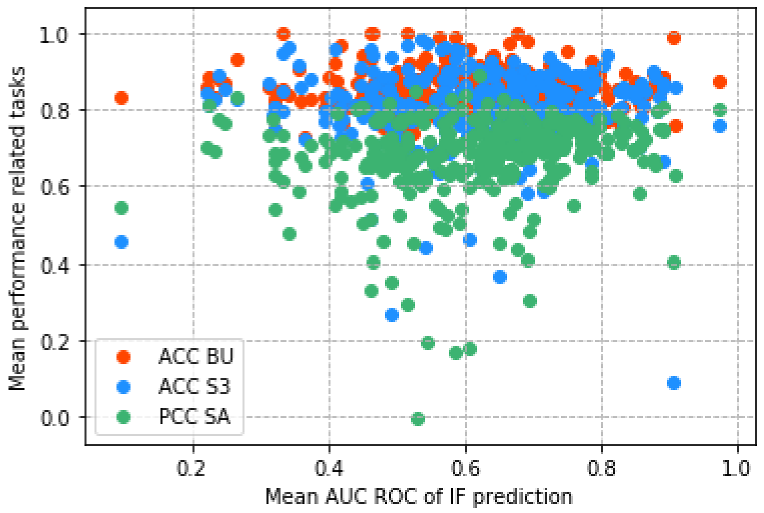

Supplementary Fig. 9. Error analyse on the individual proteins of the test set after training the IFBUS3SA model on the PPI_extendedSFD dataset including only one tenth of the PPI interface information. The mean accuracy of the prediction of the secondary structural component and buried residues, and the mean pearson correlation coefficient of the absolute solvent accessibility are plotted against the mean AUC ROC score of the PPI interface prediction per protein.

Algorithm 1 Creating buried labels based on the fraction of the accessible solvent area (ASA) over the maximum solvent accessibility (MSA) of the corresponding amino acid.

procedure ASSIGNBURIED
  for all $s \in sequences$ **do**
    for all $r \in residues(s)$ **do**
      $MSA_r \leftarrow MSA(AA_r)$
      if $ASA_r/MSA_r < 0.07$ **then**
        $BU_r \leftarrow true$
      else
        $BU_r \leftarrow false$
      end if
    end for
  end for
end procedure

Algorithm 2 Calculates the loss provided vectors of lenth N for the $labels\_mask, labels, predictions$ and $weights$ of a prediction task.

function MASKEDLOSS($labels\_mask, labels, predictions, weights$)
  $indices \leftarrow 0 \dots N$
  $keep \leftarrow indices[masking\_labels] = false$
  $labels\_selected \leftarrow labels[keep]$
  $predictions\_selected \leftarrow predictions[keep]$
  $weigths\_selected \leftarrow weights[keep]$
  $loss \leftarrow CrossEntropyLoss(labels\_selected, predictions\_selected, weights\_selected)$
  return $loss$
end function
